# Supplementary material for: Profile of Small RNAs, vDNA Forms and Viral Integrations in Late Chikungunya Virus Infection of Aedes albopictus Mosquitoes
Source: Viruses. 2021 Mar 25;13(4):553. doi: 10.3390/v13040553 (PMC8066115; doi:10.3390/v13040553)

Supplemental figure 3 . Venn diagram representing the intersection of the sRNA differential abundance across each transcript based on the three different library sizes for normalization (i.e. 1- the total number of sRNA reads that mapped on the reference genome, 2- the total number of reads analysed in edgeR (i.e. following size-filtering and counts), 3- the fraction of miRNA sized molecules that mapped onto a set of validated miRNAs). A) siRNA abundance across in infected vs blood-fed ovaries. B) siRNA abundance across in infected vs blood-fed carcasses. C) piRNA abundance across in infected vs blood-fed ovaries. D) piRNA abundance across in infected vs blood-fed carcasses.

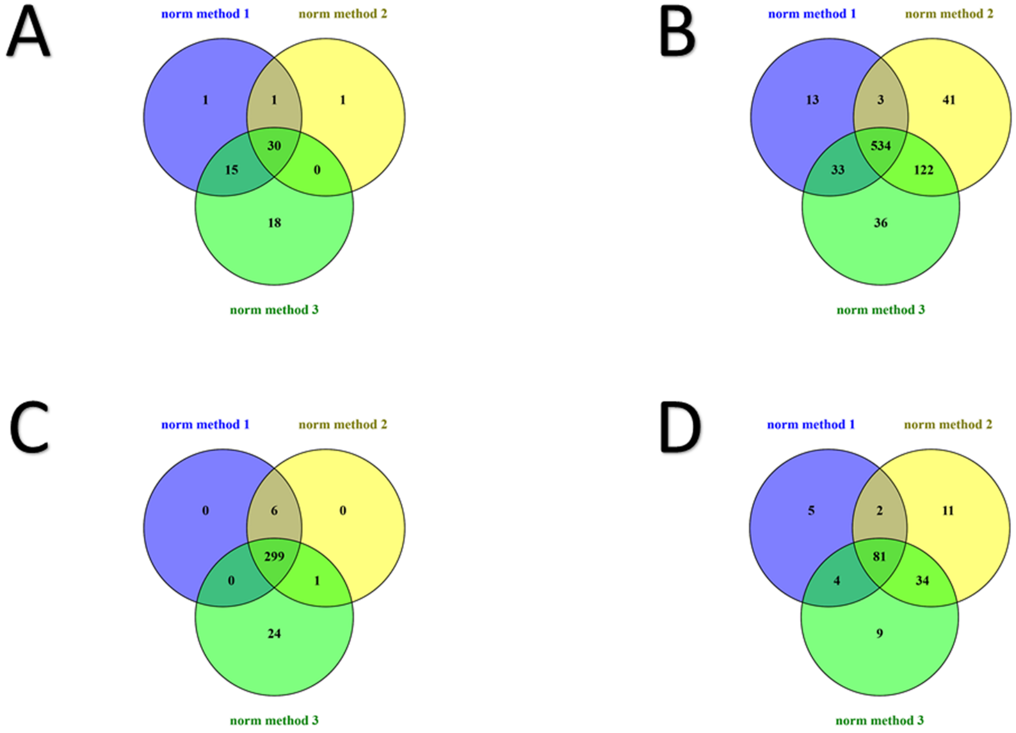

Supplement: Supplementary file 1 [file viruses-13-00553-s001.zip › SupportingInformation/Supplemental figure 3.pdf]
