# Supplementary material for: Profile of Small RNAs, vDNA Forms and Viral Integrations in Late Chikungunya Virus Infection of Aedes albopictus Mosquitoes
Source: Viruses. 2021 Mar 25;13(4):553. doi: 10.3390/v13040553 (PMC8066115; doi:10.3390/v13040553)

Supplemental figure 1. A) Scheme of the infection experiments. Colour code: grey = infection experiments; blue = blood-fed controls. B) Strategy used to study the small RNA profile of nrEVEs. To test how individual small RNAs are expressed, single molecules were counted and referred to all nrEVEs they mapped (level 1). To analyse the total small RNA profile of each nrEVE, all the small RNA molecules mapping within the boundaries of individual nrEVEs were considered (level 2)

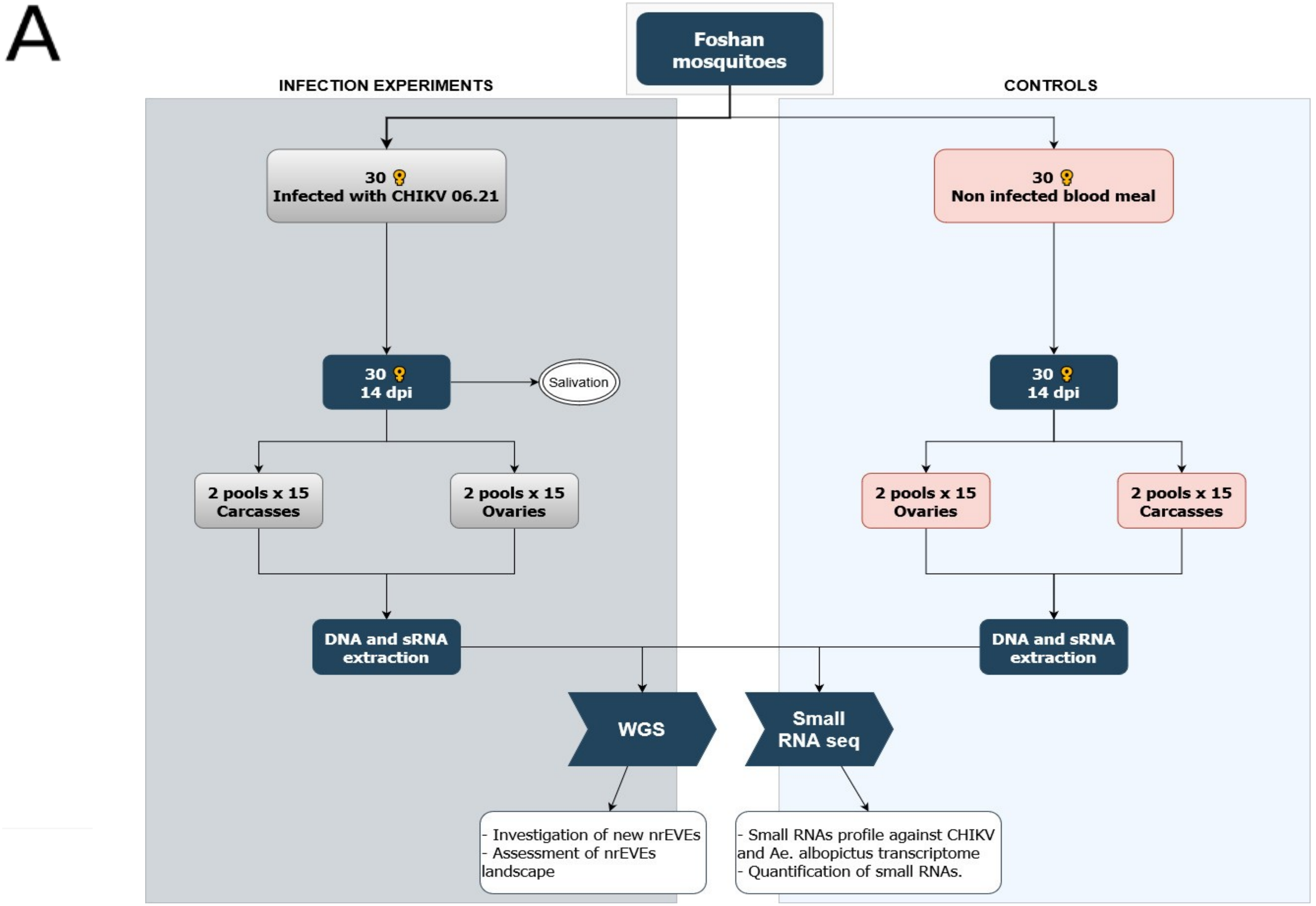

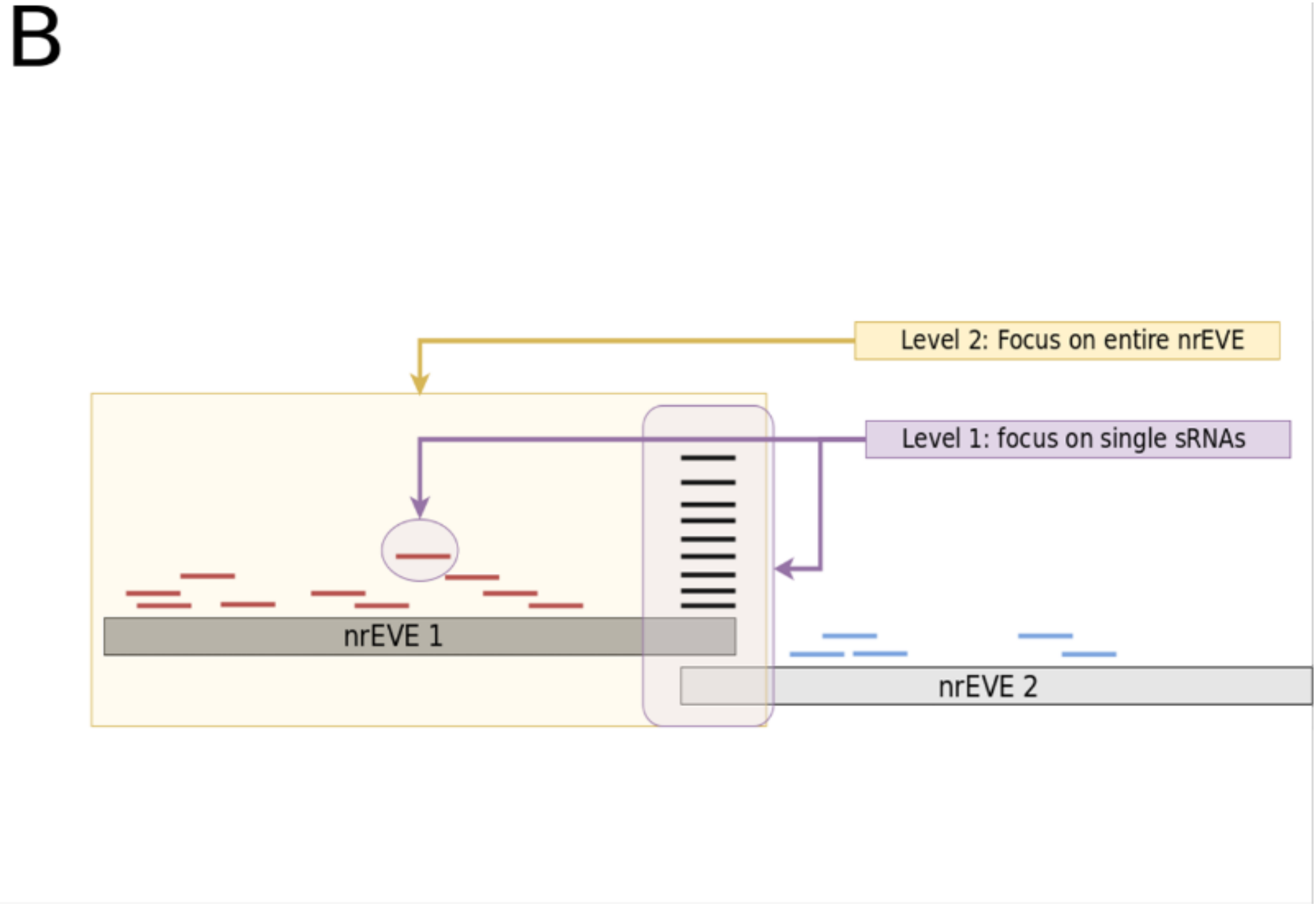

Supplement: Supplementary file 1 [file viruses-13-00553-s001.zip › SupportingInformation/Supplemental figure 1.pdf]
