# Supplementary material for: Profile of Small RNAs, vDNA Forms and Viral Integrations in Late Chikungunya Virus Infection of Aedes albopictus Mosquitoes
Source: Viruses. 2021 Mar 25;13(4):553. doi: 10.3390/v13040553 (PMC8066115; doi:10.3390/v13040553)

Supplemental figure 5. Bar plot of significantly enriched pathways based on differential sRNA abundance in CHIKV -infected ovaries and carcasses. Results refer to the Aedes aegypti KEGG pathway annotation.

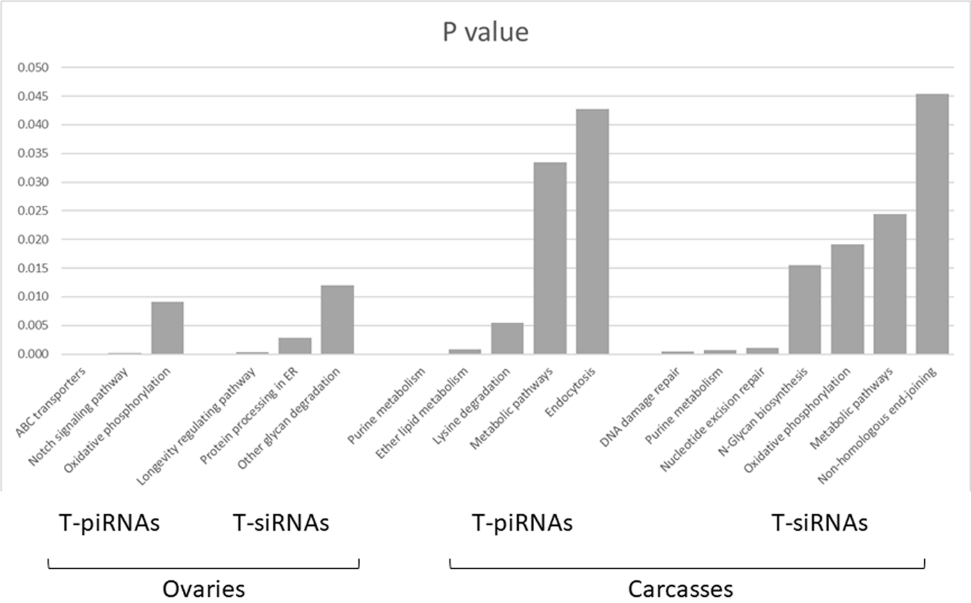

Supplement: Supplementary file 1 [file viruses-13-00553-s001.zip › SupportingInformation/Supplemental figure 5.pdf]
