# Supplementary material for: Profile of Small RNAs, vDNA Forms and Viral Integrations in Late Chikungunya Virus Infection of Aedes albopictus Mosquitoes
Source: Viruses. 2021 Mar 25;13(4):553. doi: 10.3390/v13040553 (PMC8066115; doi:10.3390/v13040553)

Supplemental figure 7. Venn diagram representing the intersection of the sRNA differential abundance across each nrEVE based on the three different library sizes for normalization. A) piRNA abundance across in infected vs blood-fed ovaries; B) siRNA abundance across in infected vs blood-fed ovaries; C) siRNA abundance across in infected vs blood -fed carcasses. No significant differential abundance was found for piRNAs in carcasses.

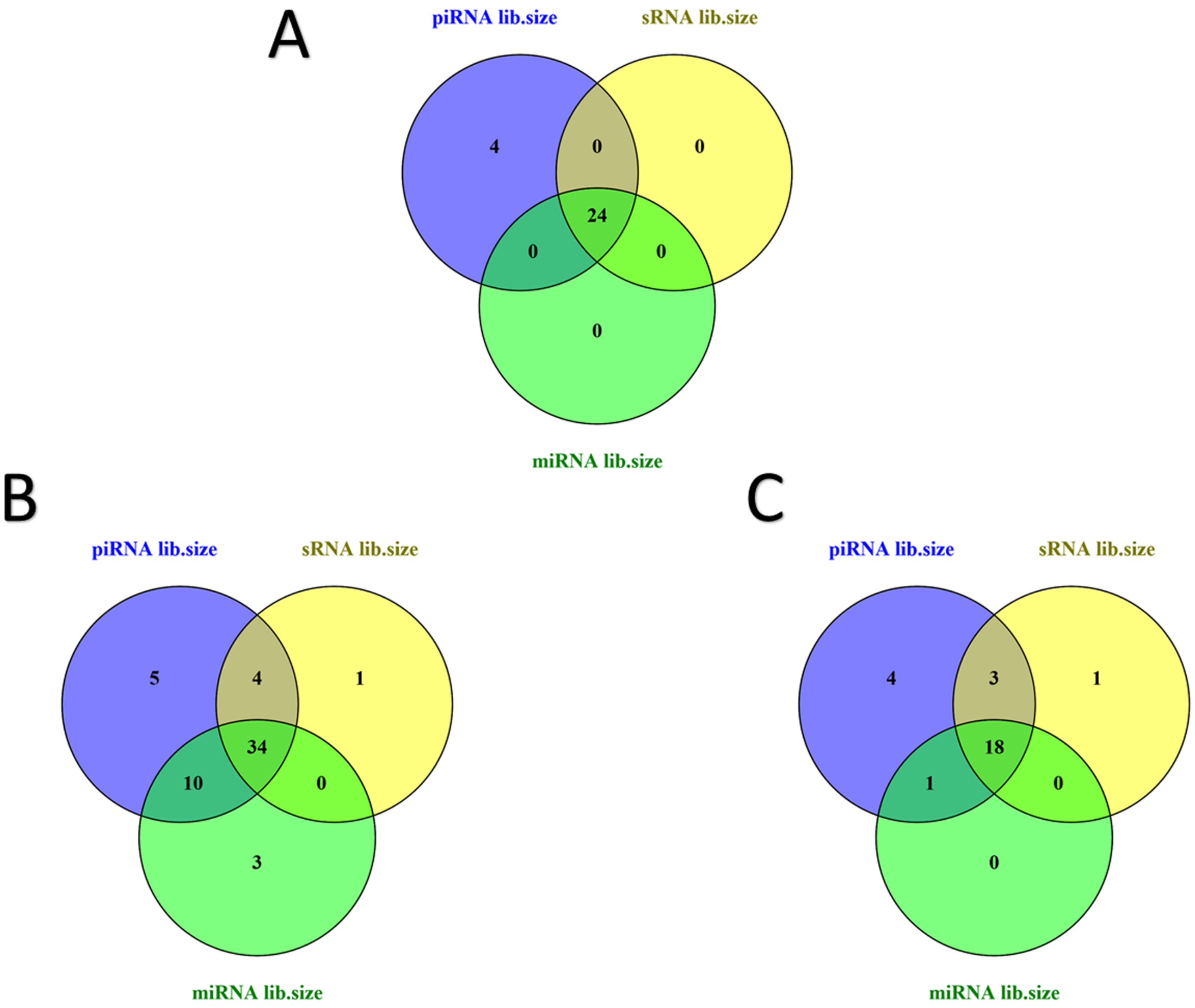

Supplement: Supplementary file 1 [file viruses-13-00553-s001.zip › SupportingInformation/Supplemental figure 7.pdf]
