# Supplementary material for: Profile of Small RNAs, vDNA Forms and Viral Integrations in Late Chikungunya Virus Infection of Aedes albopictus Mosquitoes
Source: Viruses. 2021 Mar 25;13(4):553. doi: 10.3390/v13040553 (PMC8066115; doi:10.3390/v13040553)

Supplemental figure 6. Venn diagram of the transcripts displaying differential abundance across conditions.

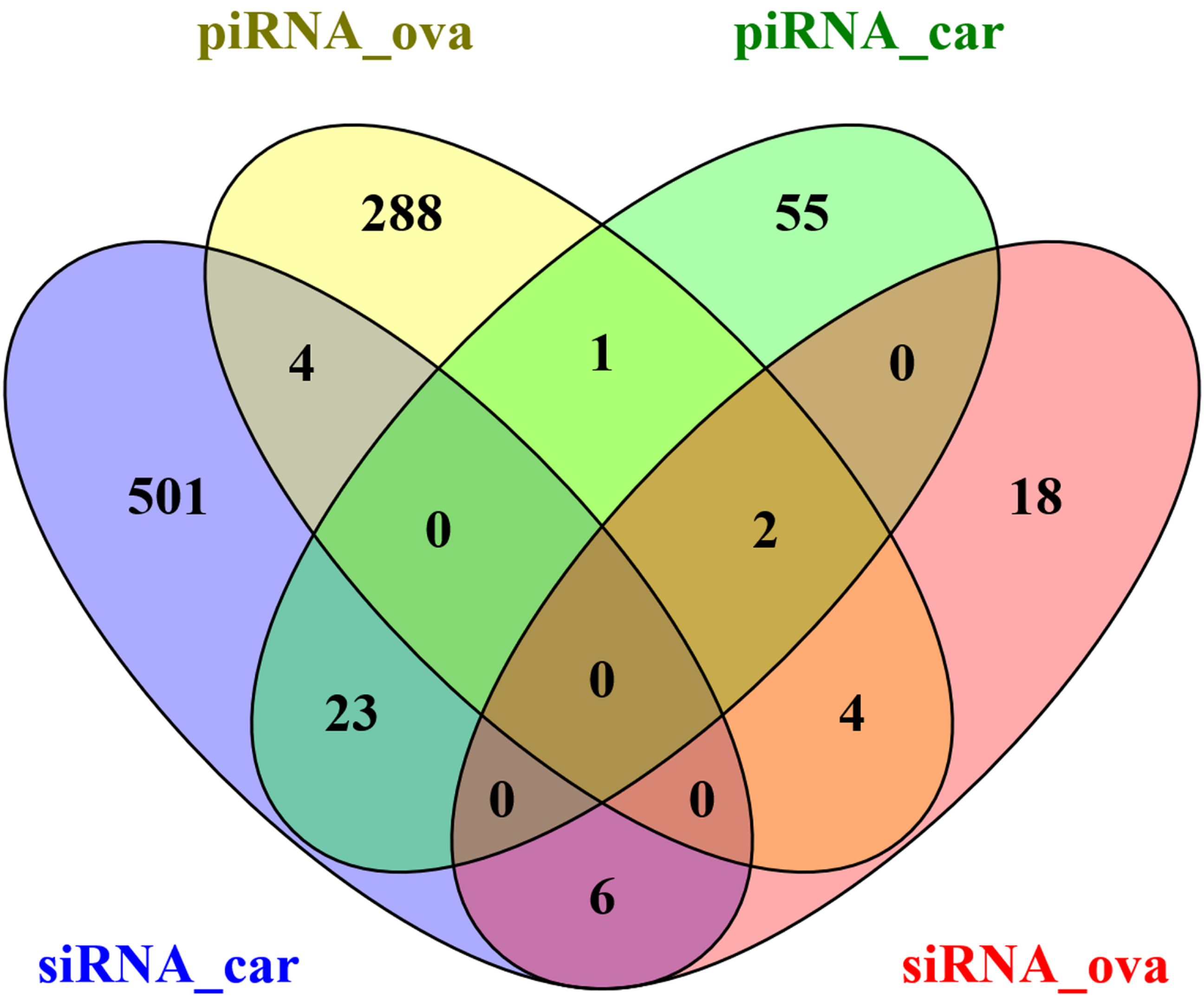

Supplement: Supplementary file 1 [file viruses-13-00553-s001.zip › SupportingInformation/Supplemental figure 6.pdf]
