# Supplementary material for: Profile of Small RNAs, vDNA Forms and Viral Integrations in Late Chikungunya Virus Infection of Aedes albopictus Mosquitoes
Source: Viruses. 2021 Mar 25;13(4):553. doi: 10.3390/v13040553 (PMC8066115; doi:10.3390/v13040553)

Supplemental figure 4. Hairpin predictions computed with miRNAFold. Left: Window between 4373-4453 of the CHIKV 06.21 genome. Candidate small RNA sequence highlighted in green. Right: Two windows (4436-4556 and 4503-4580) for a same candidate viral miRNA sequence.

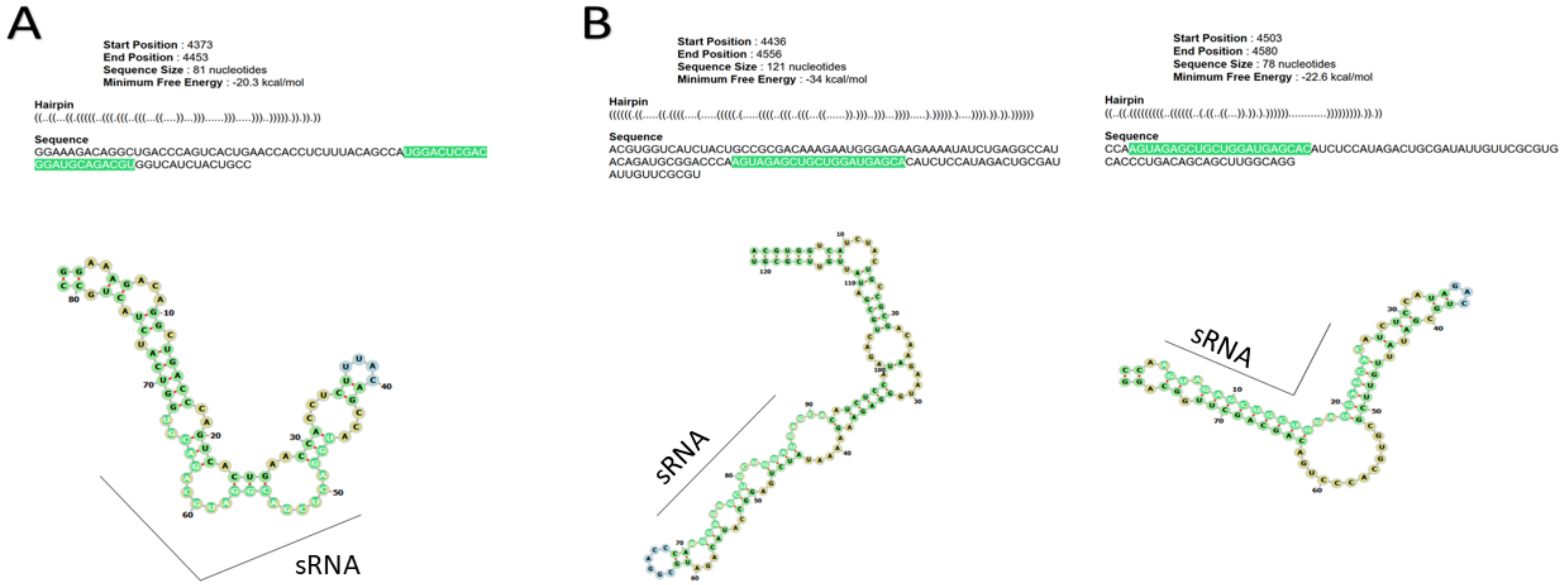

Supplement: Supplementary file 1 [file supplemental figure 4.pdf › SupportingInformation/supplemental figure 4.pdf]
